# Impact of chest subcutaneous fat on the occurrence of central venous port-related infectious complications in cancer patients

**DOI:** 10.1007/s00520-021-06109-9

**Published:** 2021-03-10

**Authors:** Jumpei Shibata, Hidetaka Kawamura, Kazuhiro Hiramatsu, Michitaka Honda, Yoshihisa Shibata, Taro Aoba, Masahiro Fujii, Atsuki Arimoto, Akira Ito, Kenta Ishii, Kojiro Omiya, Mariko Asai, Takuya Arakawa, Hirotake Gonda, Shuhei Asai, Takuya Hasegawa, Kento Kawashima, Takehito Kato

**Affiliations:** 1 Department of General Surgery, Toyohashi Municipal Hospital, 50 Aza Hachiken Nishi, Aotake–Cho, Toyohashi, Aichi 441-8570 Japan; 2 Department of Minimally Invasive Surgical and Medical Oncology, Fukushima Medical University, 7-115 Yatsuyamada, Koriyama, Fukushima, 963-8563 Japan

**Keywords:** Computed tomography, Subcutaneous fat area, Implantable central venous port, Infectious complication

## Abstract

**Purpose:**

There is no concrete evidence to support the association between the amount of subcutaneous fat area (SFA) in the central venous port-insertion site (precordium) and port-related complications. We aimed to investigate the relationship between SFA in the midclavicular line and postoperative infectious complications in patients undergoing port-insertion surgery.

**Methods:**

This was a single-institute and historical cohort study of 174 patients who underwent first central venous port implantation surgery for chemotherapy between January 2014 and December 2018. SFA in the midclavicular line was measured using preoperative computed tomography scans. The patients were divided into three groups according to SFA amount tertiles, and we investigated the association of SFA with infectious and all-cause complication events within 1 year.

**Results:**

Within a median follow-up of 306 days, the patients with intermediate SFA had significantly higher infection-free survival than those with low and high SFA (low vs. intermediate vs. high: 80.4% vs. 97.7% vs. 83.4%, respectively, *p*=0.034). In contrast, there was no significant difference in the overall complication-free survival among the groups (low vs. intermediate vs. high: 80.4% vs. 88.9% vs. 81.8%, respectively, *p*=0.29). Low SFA was independently associated with high risk of infectious complications (hazard ratio, 9.45; 95% confidence interval, 1.07–83.22, *p*=0.043).

**Conclusion:**

Low SFA in the midclavicular line was an independent risk factor for infectious complications in the chemotherapy setting. This practical indicator can be useful for optimizing patients’ nutritional status and when considering other types of vascular access to support administration of intravenous chemotherapy.

## Introduction

The central venous (CV) port system has become an essential device that facilitates long-term administration of chemotherapy. The CV port is completely implantable and enables the administration of various anticancer agents with lower risk of extravasation. Thus, patients report higher satisfaction and lower anxiety [1]. However, despite their apparent benefits, CV ports can also be a source of cancer treatment-related morbidity, such as port-related infections [2]. Cancer patients who undergo chemotherapy are at a particularly higher risk of CV port-related infectious complications owing to their inherent cancer-related characteristics, including malnutrition and immune deficiency. CV port-related infectious complications can delay chemotherapy and indirectly lead to disease progression. Thus, identifying risk factors for CV port-related infectious complications is important in the management of cancer patients.

Cancer patients with malnutrition can be susceptible to CV port-related infectious complications because of their little subcutaneous fat in the surgical site, which may induce dermal necrosis and eventual exposure of the CV port. The close distance between the device and the outer skin may contribute to easy invasion of pathogens. Although surgeons believe that low subcutaneous fat in the port-insertion site might be a potential risk factor for port-related infectious complications, no study has reported an association between the subcutaneous fat area (SFA) and port-related infection.

Generally, body mass index (BMI) is used as an indicator of nutritional status and an index of obesity. However, the BMI does not specifically reflect the actual body’s composition of muscle, visceral adipose, and subcutaneous adipose tissues [3]. In comparison, computed tomography (CT)–derived analytic imaging of body composition may lead to a more specific assessment of infection risk at the operative site [4]. More specifically, in CV port insertion, this objective measurement may potentially reflect the surgeons’ preoperative subjective assessment of the operative site of the SFA in the midclavicular line. Within this context, we speculate that applying these objective surgical site assessment measures might inform preoperative risk evaluation and contribute to clinical decision-making.

We aimed to evaluate the association between subcutaneous fat in the midclavicular line and port-related infectious complications in adult patients undergoing CV port insertion for chemotherapy. We hypothesized that low SFA would be an independent risk factor for CV port-related infectious complications.

## Patients and methods

### Study design and participants

**Fig. 1:**
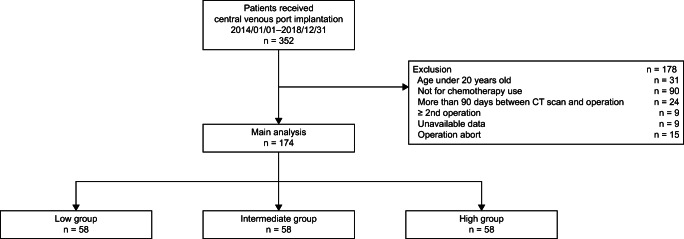
Patient selection flowchart

This was a single-center, historical cohort study of patients who underwent their first CV port implantation surgery between January 2014 and December 2018. The exclusion criteria were age under 20 years, CV port implantation for non-chemotherapy purposes, no CT scan within 90 days before CV port implantation, missing data, and multiple CV port insertions during the study period. We excluded minors younger than 20 years since we sought a study population of adults with similar cancers and treatment regimens. We defined the age of 65 years as the cut-off, since persons over that age are categorized as “elderly” in our country. Figure 1 shows the patient enrollment flowchart.

This study was approved by the Institutional Review Board of Toyohashi Municipal Hospital and was conducted in accordance with the ethical standards of the respective committees on human experimentation (institutional and national) and the 1964 Helsinki Declaration and its later amendments. Consent to participate in the study was obtained using an opt-out method.

### Measurement and assessment of SFA at the midclavicular line

**Fig. 2:**
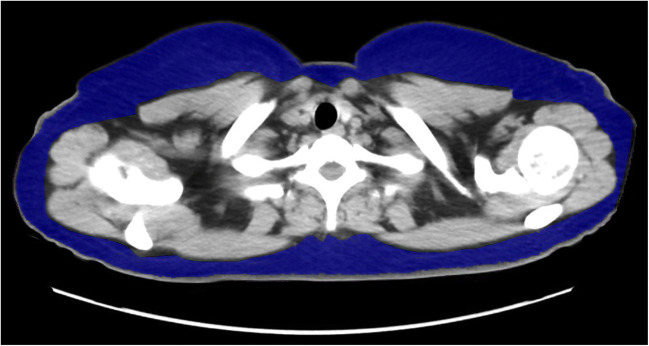
A typical image used to measure the subcutaneous fat area in the midclavicular line using multi-detector computed tomography data. Regions in blue indicate the subcutaneous fat area

Patients underwent CT within 90 days before the procedure using a 64-row multi-detector scanner (Aquilion 64; Canon Medical Systems, Tochigi, Japan) with them positioned in the decubitus dorsalis position. The reason for defining 90 days as the maximum time window for the pre-procedural CT scan is that the judgment regarding response or progression of cancer is usually performed within 90 days. The images obtained were transferred to a workstation (SYNAPSE VINCENT; FUJIFILM, Tokyo, Japan), wherein SFA at the level of the umbilicus was utilized to measure the midclavicular level semi-automatically (Fig. 2). The subsequent calculation process was as follows: first, the researcher manually adjusted the height of the axial CT scan at the midclavicular line. Second, the workstation semi-automatically detected and calculated the SFA according to the Hounsfield scale (−50 to −100 Hounsfield units). Third, the researcher readjusted the area manually if the semi-automatically detected area was obviously wrong.

The patients were divided into three groups, low, intermediate, and high, according to the SFA amount tertiles in the midclavicular line.

### Data collection and outcomes

The primary outcome measure was the incidence of CV port-related infectious events that required port removal within 1 year after the initial placement. The secondary outcome measures were all-cause events that required port removal. The occurrence of clinical events was determined from inpatient/outpatient medical records. All follow-up data within 1 year after the initial placement were collected. Patients who did not experience any events or died were censored on the date of the final observation. Other relevant clinical data, including age, sex, BMI, Charlson comorbidity index (CCI), American Society of Anesthesiologists performance status (ASA-PS), lymphocyte count, serum albumin level, site of malignancy, grade of surgeon, port-insertion vessel, and the side of port-insertion, were also obtained from the electronic medical records.

### Statistical analyses

Categorical data were expressed as frequencies and percentages and compared between groups using the chi-squared test. Non-normally distributed variables were presented as medians (interquartile range [IQR]) and compared between groups using the Kruskal-Wallis test. Event-free survival curves were constructed using the Kaplan-Meier method and compared using the log-rank test. Univariate and multivariate Cox proportional hazard models were used to calculate hazard ratios (HRs) and 95% confidence intervals (CI) for the outcomes. All statistical analyses were conducted using R version 3.5.1 (R Foundation for Statistical Computing, Vienna, Austria). A *p* value <0.05 was considered statistically significant.

## Results

### Baseline patient characteristics

Of the 352 patients who underwent CV port implantation, 174 were included in the analysis. In total, 58 (33.3%) patients were assigned to each SFA group. The baseline patient characteristics according to the SFA are listed in Table 1. The median (IQR) SFA was 18.2 (6.3–29.0), 60.0 (51.2–68.9), and 118.8 m^2^ (93.5–150.0) in the low, intermediate, and high SFA groups, respectively. The low-SFA group included more older adults as well as more individuals with gastrointestinal cancers. Individuals in this group also had a lower BMI. There were no significant differences in other baseline characteristics, including CCI, ASA-PS, serum lymphocyte counts, serum albumin level, grade of surgeon, access side, and access site.

**Table 1:** Patients characteristics

		Low SFA group		Intermediate SFA group		High SFA group		*p* value
		(*n*=58)		(*n*=58)		(*n*=58)		
Age in years, *n* (%)		66	(61.5–71.8)	67	(59.5–72.8)	62	(56.0–67.8)	0.019
	<65	27	(46.6%)	20	(34.5%)	33	(56.9%)	0.053
	**≥**65	31	(53.4%)	38	(65.5%)	25	(43.1%)	
BMI (kg/m^2^), *n* (%)								
	<18.5	25	(43.1%)	6	(10.3%)	0	(0.0%)	<0.001
	18.5–24.9	33	(56.9%)	45	(77.6%)	17	(29.3%)	
	25–29.9	0	(0.0%)	7	(12.1%)	30	(51.7%)	
	**≥**30	0	(0.0%)	0	(0.0%)	11	(19.0%)	
Sex, *n* (%)								
	Male	43	(74.1%)	29	(50.0%)	15	(25.9%)	<0.001
	Female	15	(25.9%)	29	(50.0%)	43	(74.1%)	
CCI, *n* (%)								
	0	29	(50.0%)	19	(32.8%)	22	(37.9%)	0.075
	1, 2	25	(43.1%)	34	(58.6%)	25	(43.1%)	
	≥3	4	(6.9%)	5	(8.6%)	11	(19.0%)	
Performance status (ASA), *n* (%)								
	I	17	(29.3%)	21	(36.2%)	18	(31.0%)	0.56
	II	22	(37.9%)	24	(41.4%)	28	(48.3%)	
	III	16	(27.6%)	12	(20.7%)	12	(20.7%)	
	IV	3	(5.2%)	1	(1.7%)	0	(0.0%)	
Lymphocyte count (/μL), *n* (%)								
	>1500	18	(31.0%)	20	(34.5%)	24	(41.4%)	0.50
	**≤**1500	40	(69.0%)	38	(65.5%)	34	(58.6%)	
Serum albumin (g/dL), *n* (%)								
	**≥**3.5	31	(53.4%)	30	(51.7%)	37	(63.8%)	0.37
	**≤**3.4	27	(46.6%)	28	(48.3%)	21	(36.2%)	
Site of malignancy, *n* (%)								
	Gastrointestinal	33	(56.9%)	30	(51.7%)	24	(41.4%)	0.0058
	Pancreas	14	(24.1%)	14	(24.1%)	7	(12.1%)	
	Gynecologic	5	(8.6%)	3	(5.2%)	7	(12.1%)	
	Hematological	2	(3.4%)	8	(13.8%)	5	(8.6%)	
	Other	4	(6.9%)	3	(5.2%)	15	(25.9%)	
Grade of surgeon, median (IQR)		3	(2–3)	3	(3–3)	3	(3–3)	0.20
Access side, *n* (%)								
	Right	56	(96.6%)	52	(89.7%)	51	(87.9%)	0.23
	Left	2	(3.4%)	6	(10.3%)	7	(12.1%)	
Access site, *n* (%)								
	Internal jugular	53	(91.4%)	55	(94.8%)	58	(100.0%)	0.10
	Subclavicular	5	(8.6%)	3	(5.2%)	0	(0.0%)	
Subcutaneous fat area on midclavicular line (m2)		18.2	(6.3–29.0)	60.0	(51.2–68.9)	118.8	(93.5–150.0)	<0.001

*ASA*, American Society of Anaesthesiologists; *BMI*, body mass index; *CC*, Charlson comorbidity index; *IQR*, interquartile range

### Short- and long-term outcomes

The short-term and long-term complications are listed in Table 2. The median follow-up period was 306 days (IQR, 167–590). During the follow-up period, 26 patients experienced the following long-term clinical events: 19 patients, infection; 2 patients, port dislocation; and 5 patients, port obstruction. The intermediate group exhibited a low rate of infection. The incidence rate of complications per 1000 catheter days was 0.51, 0.19, and 0.4 in the low, intermediate, and high SFA groups, respectively. In terms of short-term complications, such as arterial puncture, pneumothorax, or external jugular vein injury, there were no statistically significant differences among the groups.

**Table 2:** Short- and long-term outcomes

		Low SFA group		Intermediate SFA group		High SFA group		*p* value
		(*n*=58)		(*n*=58)		(*n*=58)		
Operation time (min), median (IQR)		36.5	(30.3–45.0)	36.5	(31.0–46.0)	39.5	(34.0–49.8)	0.14
Blood loss (mL), median (IQR)		0	(0–0)	0	(0–0)	0	(0–0)	0.16
Incidence rate of complications per 1000 catheter days		0.51		0.19		0.4		
Complications, *n* (%)								
	All complications	10	(17.2%)	5	(8.6%)	11	(19.0%)	0.25
	Dislocation	1	(1.7%)	1	(1.7%)	0	(0.0%)	1.0
	Infection	9	(15.5%)	1	(1.7%)	9	(15.5%)	0.023
	Obstruction	0	(0.0%)	3	(5.2%)	2	(3.4%)	0.37
Short-term complications, *n* (%)								
	Yes	1	(1.7%)	2	(3.4%)	0	(0.0%)	0.77
	Artery puncture	1	(1.7%)	0	(0.0%)	0	(0.0%)	1.0
	Pneumothorax	0	(0.0%)	1	(1.7%)	0	(0.0%)	1.0
	External jugular vein injury	0	(0.0%)	1	(1.7%)	0	(0.0%)	1.0

*IQR*, interquartile range

### Association between SFA in the midclavicular line and long-term clinical outcomes

**Fig. 3:**
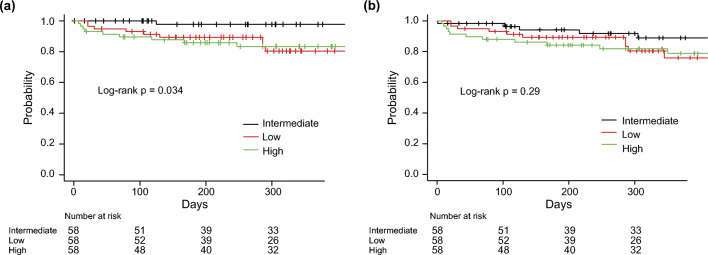
Kaplan-Meier analysis of a infectious complication events and b all complication events according to the amount of subcutaneous fat area

The Kaplan-Meier curves for infectious and all-cause complications are shown in Fig. 3. Within a median follow-up period of 306 days, the intermediate SFA group showed a significantly higher infection-free survival (low vs. intermediate vs. high: 80.4% vs. 97.7% vs. 83.4%, respectively, *p*=0.034). In contrast, there was no significant difference in the overall complication-free survival among the three groups (low vs. intermediate vs. high: 80.4% vs. 88.9% vs. 81.8%, respectively, *p*=0.29).

The results of the Cox regression analysis for cumulative incidence of infectious complications are shown in Table 3. In the univariate analysis, the HRs for infectious complications were 9.00 (95% CI: 1.14–71.04, *p*=0.037) and 9.08 (95% CI: 1.15–71.66, *p*=0.036) for the low and high SFA groups, respectively. After adjusting for BMI, age, and sex, low SFA was independently associated with a higher risk of infectious complications, with an HR of 9.45 (95% CI: 1.07–83.22, *p*=0.043).

**Table 3:** Multivariate Cox proportional hazard model for infectious complications

Variables		Unadjusted			Adjusted		
		HR	95% CI	*p* value	HR	95% CI	*p* value
Subcutaneous fat area on midclavicular line							
	Intermediate group	(Reference)			(Reference)		
	Low group	9.00	(1.14–71.04)	0.037	9.45	(1.07–83.22)	0.043
	High group	9.08	(1.15–71.66)	0.036	4.96	(0.47–52.06)	0.18
BMI							
	<18.5	(Reference)			(Reference)		
	18.5–24.9	0.81	(0.22–3.08)	0.76	1.24	(0.30–5.06)	0.77
	25–29.9	1.35	(0.32–5.65)	0.68	2.50	(0.25–24.82)	0.44
	≥30	2.87	(0.58–14.24)	0.20	4.76	(0.42–54.40)	0.21
Age							
	<65	(Reference)			(Reference)		
	≥65	0.49	(0.19–1.24)	0.13	0.65	(0.25–1.69)	0.37
Sex							
	Male	(Reference)			(Reference)		
	Female	0.72	(0.29–1.79)	0.48	0.68	(0.24–1.95)	0.48

Adjusted for BMI, age, sex

*BMI*, body mass index; *CI*, confidence interval; *HR*, hazard ratio

## Discussion

To date, there has been no evidence to support the association between SFA and CV port-related infections. This study showed that low SFA in the midclavicular line was significantly associated with a high incidence of infectious port-related complications within 1 year in patients who underwent CV port-insertion for chemotherapy. Evidence to date has been limited or sparse in this field. These findings are clinically significant as they substantiate the surgeons’ impression that patients who have a low fat area in the surgical site may have a high risk of infections.

Preoperative evaluation of a patient’s risk of infectious complications according to SFA will be helpful to prevent port-related complications, ensure compliance with the chemotherapy plan, and maintain a sufficient dose intensity. This study showed that low, but not high, SFA in the midclavicular line was significantly associated with a high incidence of infectious port-related complications in patients who underwent CV port-insertion for chemotherapy. Previous studies have reported the relationship between BMI and infectious events; however, these reports maintained that both low and high BMI had an impact on the risk of infections [5, 6]. This discrepancy in the results may be because BMI is a nonspecific parameter that cannot differentiate between fat mass and body lean mass or between visceral fat mass and subcutaneous fat mass. Meanwhile, the SFA in the midclavicular line is a more specific parameter than BMI for evaluating the volume of SFA at the surgical site.

In this study, the SFA at the level of the midclavicular line was utilized to semi-automatically measure the midclavicular level fat area in the surgical site. Low SFA in the midclavicular line was found to be an independent risk factor for port-related infectious events, after adjusting for BMI, in patients who underwent CV port-insertion for chemotherapy. These findings indicate that SFA in the midclavicular line is a more appropriate measure than BMI. Importantly, SFA could be a simple and useful risk indicator in patients undergoing an indwelling port procedure for chemotherapy.

SFA in the current study was measured using CT scans, which is the gold standard for evaluating cancer progression. SFA can also be measured using semi-automated methods for the assessment of body composition [3]. Therefore, SFA can be easily measured preoperatively using various modalities, including CT [7]. Previous studies have reported several risk factors for port-related infections, including hematological malignancies, pancreatic cancer, head and neck cancer, neutropenia, chronic steroid use, lack of perioperative antibiotic use, poor performance status, previous infection, parenteral nutrition, immediate palliative care after implantation, chemotherapy in the non-adjuvant setting, and surgery conducted by an inexperienced surgeon [8–12]. Some previous studies also evaluated the clinical relevance between surgical site infection and its morphometric characteristics, other than BMI, in abdominal surgeries [4, 13–15]. They suggested that a high amount of SFA increased the risk of surgical site infections. Another study indicated a relationship between low BMI and CV port complications [16]. However, these studies did not focus on the location of CV port insertion or conduct a morphometric assessment; our research focused on both topics.

Low SFA in the implantation site may not only be a risk factor for exposure of the CV port, but also suggests undernutrition, which increases the susceptibility to infection [17] and port-related complications.

This study has some limitations. First, the sample size was relatively small, which may have caused an inadequate adjustment for confounding factors and wide confidential intervals. However, we were able to adjust for the important confounders (BMI, age, sex) in the analysis of the relationship between SFA and infectious events. Additionally, we showed a significant correlation between SFA and infectious events. Second, the SFA calculation method was originally developed for measuring fat areas at the level of the umbilicus, not at the level of the midclavicular line. Therefore, the results should be interpreted cautiously until confirmed by further studies. Third, it is possible for cancer patients to show significant changes in body shape within 90 days. This can be a source of measurement bias.

However, this study provided the new finding that low SFA in the midclavicular line is significantly associated with a high incidence of infectious port-related complications in patients who underwent CV port-insertion for chemotherapy. These findings verified the surgeons’ belief that patients with low SFA have a high risk of infectious events. SFA can be conveniently measured in cancer patients using CT, which is routinely performed in this context. Further, SFA is a more specific indicator than BMI. Therefore, despite this study’s limitations, the present findings provide new insight into the risk assessment of CV port-related infections. Our research suggests that physicians should personalize the choice of vascular access to meet the patients’ treatment regimen necessities, while also seeking to reduce the risk of infection and other port-related complications in patients with low SFA. Currently, arm ports have been developed as a new approach to indwelling infusion systems. These devices are smaller and show no greater risk of harm compared with ports placed in the chest [18]. In order to reduce the risk of infection, the port indwelling site might be changed from the chest to the arm in adult patients with lower SFAs. Further studies are needed to validate the results and determine the generalizability of our findings in other populations.

### Conclusion

CT-derived SFA in the midclavicular line is an independent indicator of infectious complications in chemotherapy settings, making it a useful indicator when choosing other types of vascular access for chemotherapy administration, such as an arm port, and optimizing the patient’s nutritional status.
